# Coronavirus Disease 2019 Vaccinations in Patients With Chronic Liver Disease and Liver Transplant Recipients: An Update

**DOI:** 10.3389/fmed.2022.924454

**Published:** 2022-06-22

**Authors:** Pimsiri Sripongpun, Nawamin Pinpathomrat, Jackrapong Bruminhent, Apichat Kaewdech

**Affiliations:** ^1^Gastroenterology and Hepatology Unit, Division of Internal Medicine, Faculty of Medicine, Prince of Songkla University, Songkhla, Thailand; ^2^Department of Biomedical Sciences and Biomedical Engineering, Faculty of Medicine, Prince of Songkla University, Songkhla, Thailand; ^3^Division of Infectious Diseases, Department of Medicine, Faculty of Medicine Ramathibodi Hospital, Mahidol University, Bangkok, Thailand; ^4^Ramathibodi Excellence Center for Organ Transplantation, Faculty of Medicine Ramathibodi Hospital, Mahidol University, Bangkok, Thailand

**Keywords:** COVID-19, SARS-CoV-2, vaccine, cirrhosis, liver transplantation, immune

## Abstract

Coronavirus disease 2019 (COVID-19) is a current global pandemic associated with an increased mortality, particularly in patients with comorbidities. Patients with chronic liver disease (CLD) and liver transplant (LT) recipients are at higher risk of morbidity and mortality after severe acute respiratory syndrome coronavirus 2 (SARS-CoV-2) infection. Many liver societies have recommended that these patients should receive COVID-19 vaccinations, although there are limited studies assessing risks and benefits in this population. In addition, two doses of mRNA vaccines may not provide sufficient immune response, and booster dose(s) may be necessary, especially in LT recipients. Notably, variants of concern have recently emerged, and it remains unclear whether currently available vaccines provide adequate and durable protective immunity against these novel variants. This review focuses on the role of COVID-19 vaccinations in CLD and LT recipients.

## Introduction

Severe acute respiratory syndrome coronavirus 2 (SARS-CoV-2) is a novel coronavirus that was first reported to cause a pneumonia outbreak in Wuhan, China, in December 2019 (1) and has resulted in a global pandemic since (2). The disease caused by SARS-CoV-2 is referred to as coronavirus disease 2019 (COVID-19). As of December 19, 2021, COVID-19 has affected more than 273 million people and has a mortality rate of approximately 1.9%, resulting in a substantial global public health issue (2). Since COVID-19 vaccines were launched for general population in late 2020, the overall mortality has tended to decrease. COVID-19 vaccines were developed aim to reduce morbidity, mortality, and prevent transmission. The World Health Organization (WHO) reported that 137 and 194 vaccines have been in clinical and pre-clinical development (3). As of December 30, 2021, vaccination coverage comprises of 62% of the total population and has reached 72.8% among individuals 18 years or older in the United States (4). Patients with chronic liver disease (CLD), including non-cirrhosis diseases, e.g., non-alcoholic fatty liver disease (NAFLD) (5–8), alcoholic-associated liver disease (9), cirrhosis (10–12), and liver transplant (LT) recipients (13) are at risk of increased morbidity and mortality due to liver-related or non-liver-related complications (13). Accordingly, these patients should be prioritized for vaccination (14–16). Nevertheless, there are limited data on COVID-19 vaccines among patients with CLD and LT recipients. The purpose of this review is to discuss the impact of SARS-CoV-2 infections in patients with liver disease and to review evidence-based research on COVID-19 vaccines, particularly in CLD and LT recipients. Further, we highlight extent issues such as booster doses and variants of concern with the goal of providing a reference for vaccinations in patients with CLD and LT recipients.

## Search Strategy

We searched the literature using PubMed and Scopus on 1 March 2022 with search terms “COVID-19 vaccine and cirrhosis or liver transplant”. A total of 248 titles and abstracts were retrieved and manual reviewed the abstracts to address the studies of COVID-19 vaccine in patients with CLD and LT recipients. Additional references were reviewed from the literature bibliography to identify the relevant recent data included in this narrative review.

## Effects of SARS-CoV-2 on Patients With Liver Disease

### Patients With Cirrhosis

**FIGURE 1:**
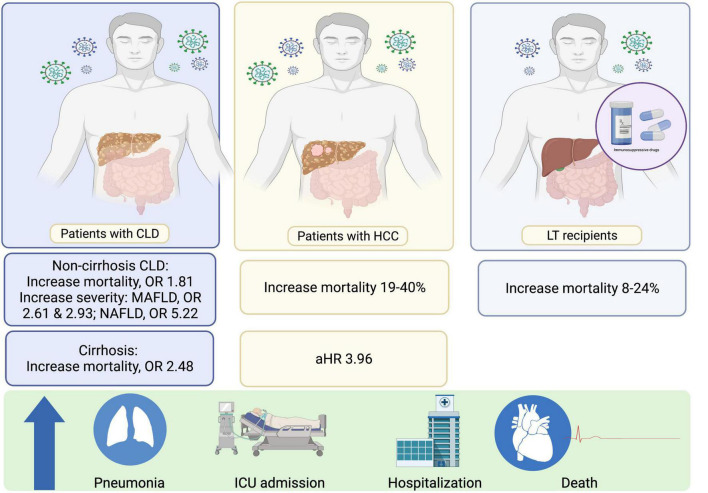
Effects of SARS-CoV-2 in patients with CLD and LT recipients. CLD, chronic liver disease; HCC, HCC, hepatocellular carcinoma; HR, hazard ratios; LT, liver transplant; MAFLD, Metabolic Associated Fatty Liver Disease; NAFLD, Non-alcoholic fatty liver disease; OR, odd ratios; SARS-CoV-2, Severe acute respiratory syndrome coronavirus 2.

Since 2020, there have been numerous reports on the prognosis and outcomes following SARS-CoV-2 infection in patients with cirrhosis. Seminal data were derived from a registry study in Asia (the APCOLIS study) (11) and combined results from the COVIDCirrhosis.org and COVID-Hep.net registries (17) (predominantly from Europe and the United States). Both registry studies reported significantly higher mortality rates in patients with cirrhosis than in control groups comprising non-cirrhotic CLD patients or patients without CLD who had SARS-CoV-2 infection, especially in patients with Child-Turcotte-Pugh B and C. A recent meta-analysis of 16 studies confirmed that in SARS-CoV-2-infected patients, the presence of cirrhosis was significantly associated with increased mortality compared to that in non-cirrhotic patients, with a pooled crude odds ratio (OR) of 2.48 (95% CI: 2.02–3.04) and a pooled adjusted OR of 1.81 (95% CI: 1.36–2.42) (18) (Figure 1). Moreover, the severity of baseline liver cirrhosis status was correlated with higher mortality (18). Cirrhosis-associated immune dysfunction (CAID) is thought to be a major contributor to poorer clinical outcomes in patients with severe liver disease (19). Notably, 25% of cirrhotic patients with SARS-CoV-2 infection only present with hepatic decompensation without any concurrent pulmonary symptoms (17). Thus, cirrhotic patients who present with any type of decompensation should be assessed for SARS-CoV-2 infection regardless of their pulmonary symptoms.

Concerns have been raised that all hospitalized cirrhotic patients generally have higher in-hospital mortality compared to patients without cirrhosis. The issue of whether SARS-CoV-2-infected cirrhotic patients have a higher risk of poor outcomes compared to cirrhotic patients hospitalized for other causes is of interest. A small retrospective study from Italy (comprising 50 SARS-CoV-2-positive and 47 SARS-CoV-2-negative patients with cirrhosis) (20) and two national-level studies from the United States (12, 21) (comprising 305 and 8941 SARS-CoV-2-positive and 3301 and 53476 SARS-CoV-2-negative patients with cirrhosis, respectively) reported that SARS-CoV-2 infection was significantly associated with higher all-cause mortality among patients with cirrhosis [adjusted hazard ratios (aHRs): 2.38–3.594]. However, several reports from matched-cohort studies in North America, Canada (22), and Korea (23) demonstrated no significant difference in overall mortality between cirrhotic patients with and those without SARS-CoV-2 infection.

Furthermore, among patients with CLD, NAFLD or the recent nomenclature, Metabolic Associated Fatty Liver Disease (MAFLD) (24), is a rising problem affecting more than one-third of people globally (25). There have been some studies evaluating the role of this particular liver disease in COVID-19 patients. Moctezuma-Velázquez et al. reported that NAFLD is the independent factor associated with the requirement of invasive mechanical ventilator and mortality in COVID-19 patients (26). Subsequently, the association between NAFLD or MAFLD and the increase COVID-19 severity has been confirmed by two meta-analyses (27, 28) (Figure 1). Hegyi et al. showed that different spectrum of the disease has a different impact on COVID-19 outcomes i.e., MAFLD significantly increased the severity of COVID-19 infection with an OR of 2.61 (95%CI: 1.75–3.91), whereas NAFLD showed an OR of 5.22 (95%CI: 1.94–14.03) (27). Similarly, Pan et al. concluded that MAFLD increased the severity of COVID-19 with a pooled OR of 2.93 (CI: 1.87–4.60) (28). In addition, MAFLD patients with high interleukin-6 levels had a higher risk of severe COVID-19 (adjusted OR 1.14, 95%CI 1.05–1.23; *P* = 0.002) (29).

### LT Recipients

The presentation of LT recipients with SARS-CoV-2 infection is distinct to that of cirrhotic patients, with fewer detrimental effects observed in LT recipients. Of note, LT recipients often share common demographic characteristics and comorbidities associated with known risk factors for unfavorable outcomes in SARS-CoV-2-infected patients. For instance, LT recipients are more likely to have renal impairment and diabetes compared to the general population. Hence, risk adjustments incorporating these factors are crucial when determining whether LT itself is a risk factor for higher mortality in patients with SARS-CoV-2 infection (19). The overall mortality of SARS-CoV-2 infection in LT recipients is approximately 20% (range: 8–24% in reports which included at least 10 LT recipients) (19, 30) (Figure 1). Despite this high percentage, LT itself is not associated with a higher mortality rate in patients with SARS-CoV-2 infection after risk adjustment (19). In SARS-CoV-2-infected LT recipients that are symptomatic, respiratory symptoms are the main presenting symptom; the difference in clinical manifestation between LT recipients and non-LT recipients is that gastrointestinal symptoms are more common and are observed in up to 42% of LT recipients (19, 30).

LT recipients are typically on immunosuppressive therapy, which may impact the course of disease in the case of SARS-CoV-2 infection. Patients on immunosuppressive agents may be more vulnerable to severe infection, and the management of immunosuppressive agents during the treatment of SARS-CoV-2 infection may be problematic. A recent systematic review and meta-analysis of 509 LT recipients with SARS-CoV-2 infection reported that the use of immunosuppressants was significantly more common in non-severe cases and in patients who survived from infection, with pooled ORs of 10.7 (95% CI: 3.11–36.94) and 16.0 (95% CI: 11.48–22.30), respectively (31). The results demonstrated that continuation of immunosuppressive agents was not harmful and may be associated with more favorable outcomes following SARS-CoV-2 infection. Therefore, withdrawal of immunosuppressants in LT recipients with COVID-19 should be discouraged. Nonetheless, distinct outcomes of different immunosuppressive therapies have been reported. A nationwide study of 111 LT recipients in Spain demonstrated that baseline immunosuppression containing mycophenolate was an independent predictor of a severe disease course, especially at doses >1,000 mg/day (32). In contrast, a multicenter study from Europe reported that tacrolimus use was independently associated with a lower mortality (33). However, these results were derived from observational studies, which precluded definitive recommendations regarding types of immunosuppressive therapy for the management of LT recipients with SARS-CoV-2 infection. Notably, although immunosuppression may not be associated with more severe disease or higher case mortality rates among patients who have already been infected with SARS-CoV-2, immunosuppression may affect immunogenicity following COVID-19 vaccination, which will be discussed later in this review.

### Patients With Hepatocellular Carcinoma

The direct impact of SARS-CoV-2 infection in patients with hepatocellular carcinoma (HCC) with regard to disease severity and mortality are not well established. To date, there have only been two small reports of patients with HCC who were infected with SARS-CoV-2, comprising 21 and 22 patients with HCC, respectively. The mortality rates in these reports varied from 19 to 40% (9, 34). The second report, which originally included 867 patients with CLD of any severity, demonstrated that among patients with CLD, decompensated cirrhosis and presence of HCC were independent predictors of mortality due to SARS-CoV-2 infection, with adjusted HRs of 2.41 (1.34–4.32), and 3.96 (1.74–8.98), respectively (9).

In different circumstances, the SARS-CoV-2 pandemic has indirectly impacted the HCC management cascade. Several studies have compared the management of HCC between 2020 and the same period in 2019 and reported a significant reduction in HCC surveillance completion rates and the number of new HCC cases (35, 36). In patients already diagnosed with HCC, delayed treatment rates were significantly higher and the overall response rate based on post-treatment imaging was lower in 2020 than in 2019 (34, 37).

## Immunogenicity of COVID-19 Vaccines

Vaccination is an effective measure to resolve a pandemic (38). Although different vaccine platforms induce distinct targets of host immunity (39–41), most vaccines induce adaptive immune responses. Vaccines induce B cells and T cells to respond specifically to a target pathogen upon re-exposure (42). These specific immune responses can be enhanced using the same or different vaccines (43). Booster vaccines operate on the selection and expansion of memory cells to increase the quantity and specificity of the immune responses against the pathogen (44, 45).

### Immune Responses to COVID-19 Vaccines

With regard to COVID-19 vaccines, the entire virus or components of the virus are used to design and construct the vaccines (46, 47). Immunodominant antigens are present on spike proteins of SARS-CoV-2, particularly on the receptor binding domain (RBD) (48, 49). The binding domain plays a crucial role in viral invasion by interaction with the angiotensin-converting enzyme 2 receptor on host cells (50). Therefore, most of the advanced vaccine platforms such as mRNA, viral vectors, and protein subunits employ this antigen for vaccine construction (41, 47, 51). After being primed and boosted by vaccines, B cells differentiate into plasma cells and secrete specific antibodies, predominantly comprising immunoglobulin G (IgG) (49). RBD-specific IgG can neutralize the virus and prevent it from attaching to host cells (52).

Antibody responses are used as the main laboratory parameter for measuring immune responses pre- and post-vaccination (53, 54). High antibody titers are used as a proxy of vaccine effectiveness (55, 56). However, neutralizing antibodies (NAs) are a more accurate measure of the effectiveness of antibodies against novel variants (57–59). Indeed, NA levels are an immune correlate of protection used to predict vaccine efficacy without conducting efficacy trials (56, 60). There are no definite thresholds for NA which can assess the clinical protection. Relative to other vaccine platforms, mRNA vaccines have been demonstrated to provide excellent antibody responses (55). Additionally, viral vector vaccines provide good antibody responses and T cell responses after one or two vaccine doses (40, 44, 61). Inactivated SARS-CoV-2 vaccines, which are a classic platform, have been reported to provide minimal antibody responses (39, 62) but can reduce disease severity (63). Different essays have been used in clinical practice to measure level and functions of the antibodies. Chemiluminescence immunoassay (CLIA) is applied to quantify the antigen specific immunoglobulins (64) while virus neutralization test (VNT) can analyze the neutralizing activity using the live virus or the surrogates (65, 66).

### Immunogenicity of COVID-19 Vaccines Among Patients With CLD and LT Recipients

**FIGURE 2:**
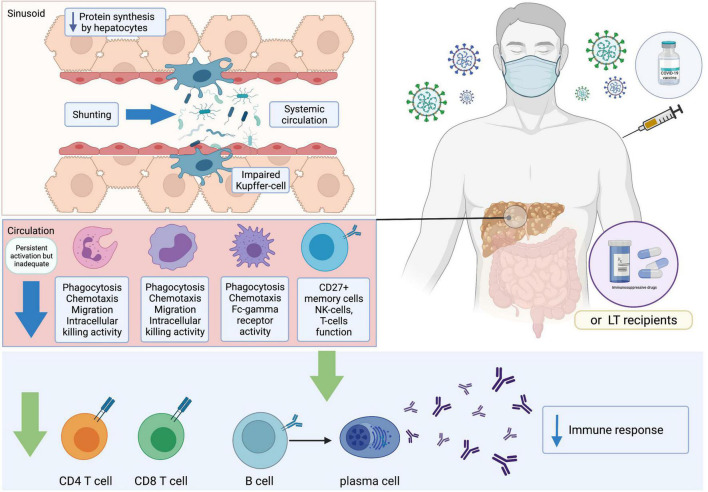
Immune responses after receiving COVID-19 vaccines. COVID-19, coronavirus disease 2019; LT, liver transplant.

CLD and cirrhotic patients typically present with impairments in immunological status (67) (Figure 2). Despite reports of the immunocompromised status of patients with chronic hepatitis B (CHB) (68), a recent study in Wuhan demonstrated that inactivated SARS-CoV-2 vaccines were safe and induced comparable immunogenicity in patients with CHB when compared to existing clinical trial data (69). Inactivated SARS-CoV-2 vaccines have also been reported to induce NAs in a large population of patients with NAFLD (70). Preliminary data have suggested that LT recipients are less likely to reach seroconversion after SARS-CoV-2 vaccination (71, 72) and antibody responses are reduced in LT patients after mRNA vaccination (72). However, the reactogenicity in LT patients is comparable with current safety data in the general population (73). To date, limited data are available on vaccines immunogenicity in patients with cirrhosis. A German study reported that seroconversion was achieved in 63% of LT recipients and 100% of cirrhotic patients and controls based on an anti-S trimer assay. However, no significant difference was observed in antibody responses between cirrhotic patients and healthy controls (74). With regard to T cell immunity, antigen-specific T cell responses against the SARS-CoV-2 spike protein were lower in patients with LT (36.6%) and cirrhosis (65.4%) than in controls (74). To date, there has been a paucity of immunogenicity studies, and immunological factors underlying inadequate vaccination responses in these populations have not been comprehensively explored. Relevant studies will be discussed in more detail in the sections on humoral-mediated immunity (HMI) and cellular-mediated immunity (CMI) in patients with CLD and LT recipients (Table 1).

**TABLE 1:** Summary of COVID-19 vaccination’ studies in patients with CLD and LT recipients.

Authors, year	Country	Patients’ group, N	Type of vaccine	Rate of antibody response by patients’ group
**Immunogenicity studies**				
Wang et al. (70) (CHESS2101)	China	NAFLD, 381	2 doses of inactivated vaccines (CoronaVac)	• Neutralizing antibodies against SARS-CoV-2 detected in 95.5% of patients. • No serious AE
Ai et al. (98) (CHESS-NMCID 2101)	China	• Non-cirrhosis, 284 • Compensated cirrhosis, 123 • Decompensated, 30 • Healthy control, 144	2 doses of inactivated vaccines	• Neutralizing antibodies against SARS-CoV-2 were lower in CLD 77.3% *vs* 90.3% in healthy (P = 0.001). • Non-cirrhotic CLD 76.8% • Compensated cirrhosis 78.9% • Decompensated cirrhosis 76.7% • Grade 3 ALT elevation in 3 patients and 1 patient related to vaccine and develop liver failure • Male sex is an independent factor of lower immune response
Thuluvath et al. (100)	United States	• Non-cirrhosis, 92 • Cirrhosis, 79 • LT recipients, 62	• 2 doses of mRNA vaccines (Moderna 47%, Pfizer 45%) or • Single dose of Johnson & Johnson vaccine (8%)	Adequate response (using Elecsys^®^ Anti-SARS-CoV-2 semi-quantitative) in • Non-cirrhosis, 69/92 • Cirrhosis, 61/79 • LT recipients, 24/62 • No serious AE • Predictors of suboptimal response: LT, use of 2-3 immunosuppressive drugs and vaccination with a single dose of Johnson & Johnson vaccine
Rabinowich et al. (72)	Israel	• LT recipients, 80 • Healthy control, 25	• 2 doses of Pfizer-BioNTech BNT162b2 SARS-CoV-2 vaccine	Adequate response^a^ (using LIAISON SARS-CoV-2 S1/S2 IgG chemiluminescent) in • LT recipients, 38/80 (47.5%) • Healthy, 25/25 • No serious AE
Timmermann et al. (101)	Germany	• LT recipients, 118	• 2 doses of mRNA vaccines or • Single dose of Johnson & Johnson vaccine	Adequate response^b^ (using Euroimmun: Anti-SARS-CoV-2 Elisa) in • LT recipients, 92/118 (78%) • No serious AE • Alcohol and MMF-based had lower response
Herrera et al. (126)	Spain	• LT recipients, 58	• 2 doses of mRNA vaccines (Moderna)	Adequate response^c^ (using Atellica [IgG and IgM]) in • LT recipients, 41/58 (71%) T-cell response (IFN-γ ELISpot ) in • LT recipients, 50/58 (86%) • No serious AE
Ruether et al. (74)	Germany	• Cirrhosis, 53 • LT recipients, 141 • Healthy control, 56	• 2 doses of mRNA vaccines or viral vector vaccine (3 in cirrhosis, 11 in LT recipient received heterologous vaccines)	Adequate response^d^ (using LIAISON [anti-S trimer], Roche Elecsys^®^ [anti-S RBD]) in • Cirrhosis, 97.9%, 100% • LT recipients, 63%, 73.9% • Healthy, 100%, 100% T-cell response in • Cirrhosis, 65.4% • LT recipients, 36.6% • Healthy, 100%
**Clinical efficacy studies**				
John et al. (94)	United States	• Cirrhosis, 20037 • Propensity-matched controls, 20037	2 doses of mRNA vaccines (Moderna 51%, Pfizer 49%)	After 28 days: receipt of 1 dose • Vaccine efficacy 64.8% • Prevent hospitalization and death 100% After 7 days: receipt of 2 doses • Vaccine efficacy 78.6% • Prevent hospitalization and death 100%
Moon et al. (91)	International countries (SECURE-Liver and COVID-Hep international reporting registries)	Confirmed SARS-CoV-2 infections • CLD, received at least 1 dose, 21 • Cirrhosis, unvaccinated, 159 • LT recipients, received at least 1 dose, 19 • LT recipients, unvaccinated, 77	Viral vector vaccines in 62%	Unvaccinated • Patients with CLD: ICU admission 20, Death 18 • LT recipients, ICU admission 7, Death 6 Receipt 1 dose • Patients with CLD: ICU admission 1, Death 0 • LT recipients, ICU admission 3, Death 2 Receipt 2 doses • Patients with CLD: ICU admission 0, Death 0 • LT recipients, ICU admission 0, Death 0 • This study highlighted the reinforcement of COVID-19 vaccination to prevent death.

*AE, adverse events; ICU, intensive care unit; Ig, immunoglobulin; LT, Liver transplantation; NAFLD, Non-alcoholic fatty liver disease; RBD, receptor binding domain; SARS-CoV-2, severe acute respiratory syndrome coronavirus 2.*

*^a^Cutoff > 15 AU/mL.*

*^b^Detectable SARS-CoV-2-specific anti-spike-protein-IgG antibodies.*

*^c^Presence of SARS-CoV-2 IgG/IgM antibodies.*

*^d^Cutoff > 33.8 binding antibody units [BAU]/mL.*

## Phase III Clinical Trials of COVID-19 Vaccine Subgroups in Patients With CLD and LT Recipients

Patients with CLD or cirrhosis and LT recipients are strongly recommended to receive vaccinations against SARS-CoV-2 to prevent severe COVID-19 and unfavorable consequences. Several SARS-CoV-2 vaccine platforms have been utilized among these populations. However, the immunogenicity, efficacy, and safety data are variable and inconsistent due to a limited number of CLD, cirrhosis, and LT patients included in phase III studies (Table 2). Only 217 (0.6%) of 37,706 participants and 196 (0.6%) of 30,351 participants received the Pfizer and Moderna vaccines, respectively (75, 76). Nevertheless, trials of the Oxford/AstraZeneca vaccine excluded patients with liver disease (except Gilbert disease) in an initial study (77), with only 1.6% of patients with liver diseases enrolled in a subsequent study (78). In addition, there is a lack of clinical data on the CoronaVac vaccine among patients with liver diseases (79). Collectively, this highlights the scarcity of data on vaccine efficacy and immune responses in cirrhotic patients who have received SARS-CoV-2 vaccines.

**TABLE 2:** Summary of widely used COVID-19 vaccines in phase III trials.

Vaccine	Dosing	Efficacy	Safety issues	Subgroup chronic liver disease (CLD)
**mRNA** **BNT162b2** (Pfizer- BioNTech) (76)	30 μg (0.3 mL) [or 10 μg (0.1 mL) in ages 5–11 years] IM × 2 doses 21 days apart Approved for ages 5 years and older (125)	95% (95.3% in those with comorbidities including CLD)	Synthetic lipid nanoparticle Contraindicated if history of severe or immediate allergic reaction to any vaccine components, including PEG	Only 217 (0.6%) of 37,706 participants Not defined severity of CLD or cirrhosis
**mRNA-1273** (Moderna) (75)	100 μg (0.5 mL) IM x 2 doses 28 days apart Approved for ages 18 and older	94.1% (Unknown in CLD patients because no vaccine or placebo patients developed COVID-19 in clinical trials)	Synthetic lipid nanoparticle Contraindicated if history of severe or immediate allergic reaction to any vaccine components, including PEG	Only 196 (0.6%) of 30,351 participants Not defined severity of CLD or cirrhosis
**AZD1222** (AstraZeneca) (78)	2 IM doses 8–12 week apart Approved for ages ≥ 18 years	74.1% (83.5% in 65 years of age and older)	The most common AEs: general pain, headache, injection-site pain and fatigue no evidence of increased overall risk of neurologic events, specifically demyelinating disease or acute transverse myelitis and thrombosis with thrombocytopenia syndrome	- Only 341 (1.6%) of 21,585 participants - Not defined severity of CLD or cirrhosis
**CoronaVac** (Sinovac) (79)	2 IM doses 3 week apart	83.5% from Turkey study	The most common AE is site-pain	Unknown in CLD patients

*AE, adverse event; CLD, chronic liver disease; COVID-19, coronavirus disease 2019; IM, intramuscular; PEG, polyethylene glycol.*

A suboptimal immune response is of concern, because suboptimal immunogenicity following vaccination against other infections in such patients is well-established and has been reported in the literature. Crucially, LT recipients who must be maintained on immunosuppressants may still be vulnerable to infection even after vaccination (80, 81). Furthermore, vaccine efficacy and safety issues still remain insufficiently defined. Only a small proportion of patients with underlying liver disease, especially those with CLD, were recruited for the BNT162b2 (0.6%), mRNA-1273 (0.6%), ChAdOx1-nCoV-19 (1.6%), and Ad26.COV2-S studies (3 out of 12 SOT recipients) (75–77, 82) (Table 2). Moreover, LT recipients considered to be immunocompromised were excluded from these studies. Therefore, a degree of hesitancy to receive vaccines among LT recipients is inevitable owing to their complex medical conditions and multiple immunosuppressants. However, COVID-19 vaccinations are strongly recommended for LT recipients based on the relative benefits compared to the risks. A survey in Italy revealed that most LT recipients accepted COVID-19 vaccines, with only a few (6%) reporting hesitancy and favorably deferring vaccination owing to concerns of unknown adverse reactions from the vaccine (83).

### mRNA-Based Vaccines

The effects of mRNA-based vaccines have been more widely evaluated in solid organ transplant (SOT) recipients. Two currently available mRNA vaccines that are widely utilized include the mRNA-1273 (Moderna) vaccine and BNT162b2 (BioNTech and Pfizer) vaccine.

An immunogenicity study using two doses of the BNT162b2 mRNA vaccine in LT recipients reported significantly weaker immune responses (72%) than that in age-matched immunocompetent individuals (94%). The geometric mean of RBD IgG and NA titers were also significantly lower among LT recipients. A combination of immunosuppressive therapy and impaired renal function were identified as risk factors for poor immune responses. Although approximately half of the participants experienced some adverse events (AEs), all AEs were mild and were more frequently observed in male participants (84). A prospective study in SOT (including LT) recipients who received the mRNA-1273 vaccine revealed discordance between HMI and CMI. CMI was elicited in approximately one-third of the patients, but no NAs were detected. CMI responses were decreased in patients who underwent kidney transplantation or those maintained on tacrolimus and prednisone regimens and more immunosuppressive drugs (85). Several predictors of poor HMI responses in LT recipients that received two doses of mRNA vaccines were reported among recipients with high tacrolimus levels, those receiving more than two immunosuppressive agents, and those with obesity. However, no significant associations were observed with recipients’ age and time from LT (86). Moderate HMI and CMI responses of the two-dose mRNA-1273 vaccine in SOT (including LT) recipients have been reported. Positive anti-RBD antibodies were detected in only one-third of the patients, and only a subset of patients produced robust NAs and CD4+ T cell responses (87).

### Adenovirus Vector-Based Vaccines

Currently available adenovirus vector-based vaccines for COVID-19 include the ChAdOx1-nCoV-19 vaccine (AstraZeneca and University of Oxford), Ad26.COV2-S vaccine (Johnson & Johnson), and Sputnik V (Gamaleya Research Institute). Immunogenicity and safety data of adenovirus vector-based vaccines in SOT recipients are limited. A small case series of 12 SOT recipients who underwent Ad26.COV2-S vaccination reported a significantly lower rate of detectable anti-RBD antibodies (17%) compared to 59% obtained with the mRNA vaccine series, which is in accordance with the trends of titer magnitude (88). Nonetheless, there has limited data in patients with CLD, only 1.6% of such patients in phase III clinical trial.

### Inactivated Vaccines

Although inactivated SARS-CoV-2 vaccines are considered safe for patients with CLD and LT recipients, results from phase III trials remain to be confirmed. Immunogenicity, efficacy, and safety data focusing on CLD and LT patients are thus limited. Among the different vaccine platforms, inactivated vaccines may offer long-term safety but with potential suboptimal immune responses. Studies on BBIBP-CoV (Sinopharm) in LT recipients are scarce. There had a cross-sectional study reported the seropositivity in kidney transplant recipients only 43% after receiving the second dose of Sinopharm vaccine (89). A prospective study that included 10 liver and 38 kidney transplant recipients receiving CoronaVac (Sinovac) revealed a significantly lower seropositivity rate compared to that in healthy individuals; moreover, antibody levels were significantly higher in patients that received BioNTech than in those that received CoronaVac (90).

### Protein Subunit Vaccines

Data regarding immunogenicity after vaccination with protein subunit vaccines are limited, and further investigations are warranted.

### Clinical Efficacy of COVID-19 Vaccines

Unvaccinated CLD and LT patients are anticipated to experience greater severity and mortality due to severe COVID-19. Vaccination is an essential tool that may alleviate these events. The efficacies of mRNA vaccines and Ad26.COV2.S were reported to be greater than 95 and 66%, respectively, in phase III studies (75, 76, 82); however, data on vaccine efficacy and clinical outcomes in CLD patients and LT recipients are limited due to the small number of participants in the studies. A recent efficacy study extracted data from the SECURE-Liver and COVID-Hep international database of 21 CLD patients that predominantly had cirrhosis and 19 LT recipients (91). Of patients with CLD, one-third were admitted to hospital, and only 5% were admitted to the intensive care unit without any fatalities. Among LT recipients, approximately the same proportion of patients underwent hospital admission; however, 16% required mechanical ventilation support, and 11% succumbed to death. All patients with severe disease received only a single vaccine dose 1–2 weeks prior. Based on these data, COVID-19 vaccination of patients with CLD and LT recipients should be encouraged to avoid unfavorable consequences (91). A single transplant retrospective study of 557 SOT (105 LT) recipients in the United States revealed that breakthrough infection after 2 weeks of full vaccination with either BNT162b2, mRNA-1273 or Ad26.COV2.S vaccines occurred in only 3 of 459 (0.65%) fully vaccinated SOT recipients. All cases were mild infections without mortality (92). A national registry in England comparing unvaccinated SOT recipients and those vaccinated with either ChAdOx1-S or BNT162b2 vaccines revealed that vaccination was not associated with a reduction in infection risk. However, vaccines offered a 20% reduction in the risk of death within 28 days after infection. Subgroup analysis revealed that both ChAdOx1-S and BNT162b2, provided protection against death with a statistically significant risk reduction was observed only in those who received ChAdOx1-S. Therefore, vaccination of SOT recipients confers some protection against SARS-CoV-2-related mortality (93). However, the efficacy of the vaccine likely depends on concurrently circulating variants of the strain in the community, and the outcomes are also subject to access to healthcare and respiratory support.

Recently, the large propensity-match controls with patients with cirrhosis study indicated that COVID-19 vaccine could prevent the hospitalization as high as 100% after obtained mRNA vaccine at least 1 dose (94). In addition, completion of 2 doses could reduce the SARS-CoV-2 infection from 64.8% (receipt of 1 dose) to 78.6% (94) (Table 1). Nevertheless, breakthrough COVID-19 infection can occur despite vaccination but not fatal. John et al. (95) reported COVID-19 vaccination can reduce the mortality of COVID-19 infection post-vaccination by approximately 80% in patients received either partial or full vaccination. Of interest, this benefit was generalized in any types of mRNA vaccine and cirrhosis severity. This study highlighted the value of COVID-19 vaccination despite the breakthrough infection can develop.

## Current Recommendations of Liver Societies

Current recommendations of renowned international liver societies indicate that patients with CLD and LT recipients should receive COVID-19 vaccines (14–16) (Table 3).

**TABLE 3:** Recommendations summary of COVID-19 vaccination in patients with CLD and LT recipients.

Society, year	Patients with CLD and HCC	Patients with LT recipients
AASLD (14)	• Should receive vaccines • Also household members and healthcare workers caring these patients should be vaccinated.	• Should receive vaccines • At least 3 months post-transplant (early as 6 weeks in highest risk for severe COVID-19) • Pre-transplant: should receive second dose 2 weeks before transplant (living donor condition) • Also household members and healthcare works caring these patients should be vaccinated.
EASL (15)	• Should receive vaccines • Also household members and healthcare works caring these patients should be vaccinated	• Should receive vaccines • 3–6 months post-transplant should be considered • Also household members and healthcare works caring these patients should be vaccinated.
AISF (16)	• Should receive vaccines, prioritized in non-cirrhotic NAFLD and ALD, cirrhosis, and HCC • Also household members and healthcare works caring these patients should be vaccinated.	• The best time is pre-transplantation in early stage (low MELD score). • The optimal time post-transplant is unknown, most center suggests 3–6 months post-transplant • Also household members and healthcare works caring these patients should be vaccinated.

*AASLD, American Association for the Study of the Liver; AISF, Associazione Italiana per lo Studio del Fegato; ALD, Alcohol-associated liver disease; CLD, Chronic liver disease; COVID-19, Coronavirus disease 2019; EASL, European Association for the Study of the Liver; HCC, Hepatocellular carcinoma; MELD, Model for End-Stage Liver Disease; NAFLD, Non-alcoholic fatty liver disease.*

### Patients With CLD and HCC

All patients with CLD and HCC are advised to receive COVID-19 vaccines. Patients should not discontinue their medications for underlying diseases including immunosuppressive agents in patients with autoimmune hepatitis (AIH). Interruption of immunosuppressants may be harmful, such as the occurrence of hepatitis flares that eventually lead to liver failure in patients with AIH. In patients with HCC who may undergo resection, locoregional treatments (such as radiofrequency ablation, microwave ablation, and transarterial chemoembolization) and systemic therapy should not be delayed due to vaccination. In this regard, vaccinations should be administered as early as possible.

### LT Recipients and Living Donors

LT recipients and living donors should also receive COVID-19 vaccines. During the pre-transplant period, patients on waiting lists for transplantation and living donors should be prioritized for vaccination (14, 16), and vaccinations should be completed within 2 weeks prior to LT (14). If vaccinations are not completed before LT, the second dose can be postponed to 6 weeks post-transplantation (14). For LT recipients who have not received the COVID-19 vaccine, the optimal timing is 3 months post-LT, as this is the timepoint of the lowest dose of immunosuppressive drugs (96). Patients that are prone to SARS-CoV-2 infection, especially patients at risk for severe COVID-19 infection, may be vaccinated at 6 weeks post-transplantation (14); however, the immune response to COVID-19 vaccines may be suboptimal.

The dose of immunosuppressants should not be reduced or discontinued during vaccination, as this may increase the risk of rejection. LT recipients with abnormal liver biochemistry tests should be assessed for acute graft rejection (14). Of note, if patients have respiratory tract symptoms despite receiving vaccines, further evaluation of SARS-CoV-2 infection is warranted. Vaccinations may be postponed in patients with an unstable medical condition or those receiving treatment with high dose immunosuppressants or corticosteroids owing to acute cellular rejection post-transplantation. Additionally, all household contacts and healthcare workers should be vaccinated against SARS-CoV-2 infection and other transmissible diseases to prevent transmission to patients (14–16, 96). Critically, other behavioral measures such as social distancing, wearing masks, and washing hands are necessary in addition to vaccinations.

## Novel Perspectives of COVID-19 Vaccines in Patients With CLD and LT Recipients

### Studies on HMI and CMI in Patients With CLD and LT Recipients

A study of non-cirrhotic patients with NAFLD [median age: 39 years, interquartile range (IQR): 33–48 years] who obtained two doses of inactivated vaccines reported that NA positivity in the serum of patients was 95.5% compared to 97.6% in the general population (97). Moreover, no serious AEs were observed, and only mild and self-limited symptoms were reported, such as injection site pain (18.4%), muscle pain (5.5%), and headache (5.2%). The CHESS-NMCID 2101 study by the same group, receipt of two doses of inactivated vaccines predominantly comprising CHB patients (87.8%), reported that NAs were detected in the same proportion of patients with CLD (76.8% in non-cirrhosis, 78.9% in compensated cirrhosis, and 76.7% in decompensated cirrhosis) (98) in contrast to the higher levels of NAs (90.3%) in healthy controls. This study highlighted the lower immune response in patients with CLD compared to that in the healthy population. Similarly, the most common side effect was pain at the injection site (8.2%). In addition, male sex was an independent predictive factor of negative serological responses to the vaccine [OR: 1.86 (1.12–3.90)]. The mechanisms underlying this effect are not fully understood but may be partly due to androgen-modulating genes associated with low antibody production (99).

**FIGURE 3:**
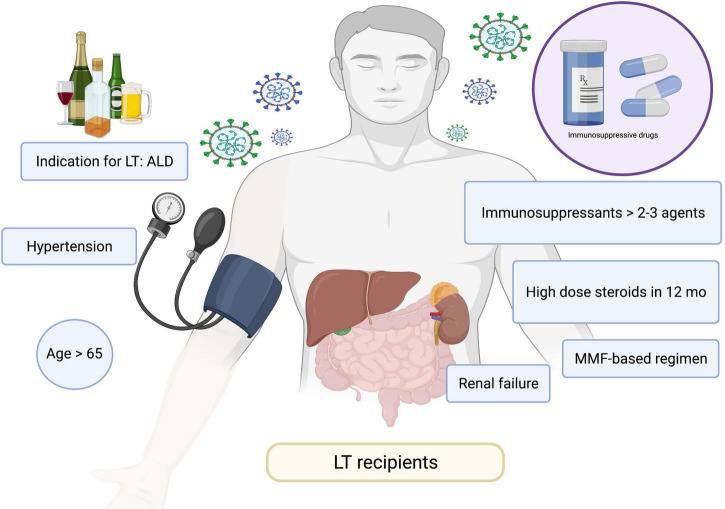
Risk factors for poor responses to COVID-19 vaccines. ALD, alcohol-associated liver disease; COVID-19, coronavirus disease 2019; MMF, mycophenolate mofetil; LT, liver transplant.

Three other prospective studies confirmed the poor antibody responses in patients with cirrhosis and even greater hyporesponsiveness in LT recipients (72, 100, 101). A study by Thuluvath et al. (100) reported that 22.8% of patients with cirrhosis and 61.3% of LT recipients exhibited poor antibody responses after receiving two doses of mRNA vaccines or a single dose of the Johnson & Johnson vaccine. Similarly, a study in Israel reported lower immune responses in patients with LT, whereby only 47.5% of patients had a positive serological response and patients had a low antibody titer (95.4 vs. 200.5 AU/mL in LT recipients and healthy control, respectively) (72). The risk factors for negative serology were older age, renal failure, receiving high-dose steroids in the last 12 months, use of mycophenolate mofetil, and use of triple immunosuppressants. A summary of the risk factors for poor vaccine responses are presented in Figure 3. A study by Timmermann et al. (101) reported lower immune responses among LT recipients. A recent study conducted in Germany reported that patients with cirrhosis had adequate antibody responses when compared to healthy individuals with 100% positivity (74). However, the T cell response was lower in cirrhotic patients compared with that in the healthy population (65% vs. 100%). Of note, LT recipients had low or absent vaccine responses in terms of both antibody and cellular responses. A summary of relevant studies is presented in Table 1.

### Booster Vaccines in Patients With CLD and LT Recipients

**FIGURE 4:**
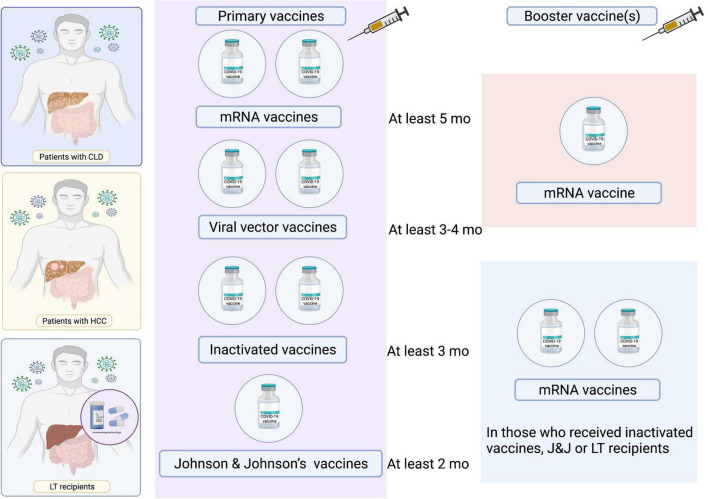
Proposed scheme of COVID-19 vaccination in patients with CLD and LT recipients. LT, liver transplant.

Immunity derived from COVID-19 vaccines decreases in parallel with the time elapsed after vaccination. Accordingly, the Centers for Disease Control and Prevention have suggested that individuals receive a booster shot at least 5 months after receiving two doses of mRNA vaccines or at least 2 months after receiving the Johnson & Johnson vaccine (102). Nevertheless, there have been limited studies in patients with CLD and LT recipients. A proposed scheme of COVID-19 vaccination in patients with CLD and LFT recipients is presented in Figure 4.

### Third Dose of mRNA-Based Vaccines

Based on several studies that have reported weak immune responses after two standard doses of mRNA-based vaccines in SOT recipients maintained on immunosuppressants, the WHO has recommended the administration of an additional dose of COVID-19 vaccines for a primary series of immunocompromised patients, including SOT recipients (103). This was supported by a study investigating immunogenicity after an additional dose of the BNT162b2 vaccine, which was offered 2 months after complete vaccination in 101 types of SOT (including the liver) recipients. The prevalence of anti–SARS-CoV-2 antibodies was increased from 40% to 68% after the third dose. Approximately 44% of seronegative patients were seroconverted after the third dose. Patients who remained seronegative were more likely to be older, maintained on a higher dose of immunosuppression, and have poor renal function. No serious AEs were reported, although allograft rejection was observed (104). Another study randomly assigned SOT recipients (including LT recipients who received two doses of the mRNA-1273 vaccine 2 months prior) to receive either a third dose of the mRNA-1273 vaccine or a placebo. This study revealed substantially higher anti-RBD antibody level responses and rates of neutralization inhibition and CMI, with minimal side effects (105).

### Fourth Dose of mRNA-Based Vaccines

A recent case series of the fourth dose of mRNA-based vaccines in 37 SOT recipients, 4 of whom were LT recipients, has been reported. Participants with a weak response (14%) and no response (84%) after three doses of the BNT162b2 vaccine were vaccinated with a fourth dose and appeared to exhibit a slightly increased HMI response; however, these effects were only observed in participants with previously developed weak immune responses. Nevertheless, no improvements were noted among participants that lacked an initial response. Additionally, the NA titers and CMI responses of both groups were low (106). Another study examined antibody responses to the fourth dose of the SARS-CoV-2 vaccine in 18 SOT recipients and reported similar results (107); in this study, 50% of participants were negative and all participants with low-positive titers were included. These findings suggest that immunogenic potential exists in poor responders, and further interventions such as monoclonal antibody administration post-exposure or temporary reduction of immunosuppressants after each vaccination may be warranted. In addition, the balance between allograft rejection and immunity protection should be taken into consideration.

Additional doses, including the third or fourth dose of the two vaccines in LT recipients, may improve vaccine immunogenicity. However, a significant proportion of patients may remain vulnerable to COVID-19. Therefore, compliance with hygiene measures and avoidance of disease exposure should be maintained. Furthermore, due to the relatively short follow-up period in previous studies, the occurrence of allograft rejection and other indirect AEs will need to be monitored in more long-term studies.

### Variants of Concern

#### Delta Variant (B.1.617.2)

The effectiveness of vaccines against the SARS-CoV-2 Delta variant has been examined for the mRNA-based vaccine (BNT162b2) produced by Pfizer Inc. and BioNTech SE and a replication-deficient simian adenovirus vector ChAdOx1 nCoV-19 (Vaxzevria) from Oxford University and AstraZeneca. The results revealed modest differences in effectiveness with the Delta variant as compared with the Alpha variant. The effectiveness of two doses of the BNT162b2 vaccine was 93.7% (95% CI: 91.6–95.3) while that of the ChAdOx1 nCoV-19 vaccine was 74.5% (95% CI: 68.4–79.4) (60). In addition, the estimated neutralization capacity of the Pfizer BioNTech vaccine against the Delta variant was 5.8-fold lower (108). The neutralizing activity against the Delta variant induced by CoronaVac (Sinovac) vaccination was lower than that due to natural infection (58).

#### Omicron Variant (B.1.1.529)

Recent data indicate that levels of NAs against the novel Omicron variant are lower, especially in viral vector-vaccinated individuals (65). Several reports have demonstrated decreased immunity against SARS-CoV-2 in long-term cohorts after vaccination (109–111). Thus, the existing immunity induced by conventional vaccination may be insufficient to protect against emerging SARS-CoV-2 variants of concern (65, 110, 112, 113).

### Safety of COVID-19 Vaccine for CLD and LT Recipients

The AEs post-vaccination usually were mild, self-limited and non-fatal including the local AEs such as pain at injection site and systemic events such as fever, fatigue, flu-like symptoms and myalgia (75–78). There have been case report and case series of acute liver injury after mRNA vaccination (114–117). Shroff et al. (114) reported 10 of 16 patients required hospitalization and therapy with good recovery. The authors concluded that some patients may experience liver injury post-vaccination from autoimmune triggering particular in who had pre-existing AIH (6 out of 16 patients). Additionally, two cases of AIH had been reported after receiving viral vector vaccines (118). None of the patients developed liver failure. At present, there has been only one case report of acute hepatic artery thrombosis from vaccine-induced prothrombotic immune thrombocytopenia that presented with acute liver failure (119). However, this is a very rare adverse event following by adenovirus vector ChAdOx1 nCoV-19 vaccination which usually occurred in younger woman. In summary, the benefit of vaccination outweighs the risk of the rare incident severe AEs.

## Future Directions and Unmet Needs

There are scarce data regarding vaccine effectiveness and immune responses including humoral and cellular immunity following COVID-19 vaccination in CLD patients and LT recipients. Challenging issues that warrant further evaluation include the effective durability of vaccine protection against SARS-CoV-2 infection, need for booster vaccines (such as fourth doses particularly in LT recipients), requirement for serologic testing to guide the need for booster vaccines, and effectiveness and safety of heterologous vaccines. Recently, variants of concern, including Omicron, have emerged as pertinent challenges. Future research is needed to verify the immunity conferred by currently available vaccines in patients with CLD and LT recipients.

Furthermore, as patients with CLD and LT recipients, considered one of the immunosuppressed populations, are at risk for developing COVID-19, the role of pre-exposure prophylaxis with monoclonal-antibody such as tixagevimab and cilgavimab in which the interim analysis showed promising results should be further investigated (120). In addition, early administration of the monoclonal antibody e.g., sotrovimab (121) and antiviral agents such as molnupiravir (122), nirmetralvir (123) may benefit in terms of prevent progression to severe COVID-19 in patients with CLD and LT recipients, nonetheless, the data in these specific groups of patients are still warrant. It is also important to note that the use of particular agents should be aware in these patients’ population e.g., remdesivir (124) should not be prescribed in those with AST/ALT above 5 times upper limit of normal, and nirmatrelvir is contraindicated in Child C cirrhosis patients (123).

## Conclusion

Patients with CLD and LT recipients constitute immunocompromised populations owing to CAID or administration of immunosuppressants. These patient populations are thus more prone to developing severe SARS-CoV-2 infection with high mortality rates when compared with the healthy population. A key preventative measure is COVID-19 vaccination, and several platforms are currently available. Several international liver societies have recommended that these patients receive vaccination owing to the greater benefits over the potential risks, despite limited studies to date. Studies have suggested that these patients are hyporesponsive to COVID-19 vaccines, and booster shots are required. In conclusion, large knowledge gaps remain regarding the effects of vaccination in patients with CLD and LT recipients, and future research is warranted. In addition to vaccination, cautionary measures such as social distancing, washing hands, and wearing masks should be practiced as part of standard lifestyle behaviors.

## Author Contributions

AK contributed substantially to the conceptualization, drafting, and critical revision of the manuscript. All authors made a substantial contribution to the literature research and drafting of the manuscript and approved the final manuscript.

## Conflict of Interest

The authors declare that the research was conducted in the absence of any commercial or financial relationships that could be construed as a potential conflict of interest.

## Publisher’s Note

All claims expressed in this article are solely those of the authors and do not necessarily represent those of their affiliated organizations, or those of the publisher, the editors and the reviewers. Any product that may be evaluated in this article, or claim that may be made by its manufacturer, is not guaranteed or endorsed by the publisher.

## Abbreviations

AASLD: American Association for the Study of Liver Diseases
ACE2: Angiotensin I Converting Enzyme 2
Ad26.COV2-S: vaccine (Johnson & Johnson)
AEs: adverse events
AIH: autoimmune hepatitis
ALD: alcohol-associated liver disease
ALT: alanine aminotransferase
APASL: Asian-Pacific Association for the Study of the Liver
BNT162b2: Pfizer-BioNTech mRNA vaccine
CAID: Cirrhosis-associated immune dysfunction
CHB: chronic hepatitis B
ChAdOx1-nCoV-19: vaccine (AstraZeneca and University of Oxford)
CI: Confidence interval
CLD: chronic liver disease
CMI: cellular mediated immunity
COVID-19: coronavirus disease 2019
EASL: European Association for the Study of the Liver
HBV: hepatitis B virus
HCC: hepatocellular carcinoma
HMI: humoral mediated immunity
HR: hazard ratios
IgG: immunoglobulin G
IM: intramuscular
IQR: Interquartile range
LT: Liver transplantation
MAFLD: Metabolic Associated Fatty Liver Disease
MELD: Model for End-Stage Liver Disease
MMF: mycophenolate mofetil
mRNA-1273: Moderna mRNA vaccine
NA: neutralizing antibody
NAFLD: Non-alcoholic fatty liver disease
OR: odd ratios
PEG: polyethylene glycol
RBD: receptor binding domain
SARS-CoV-2: Severe acute respiratory syndrome coronavirus 2
SOT: solid organ transplant Sputnik V (Gamaleya Research Institute)
WHO: World health organization.
